# Genome-wide Association Study on Platinum-induced Hepatotoxicity in Non-Small Cell Lung Cancer Patients

**DOI:** 10.1038/srep11556

**Published:** 2015-06-23

**Authors:** Songyu Cao, Cheng Wang, Hongxia Ma, Rong Yin, Meng Zhu, Wei Shen, Juncheng Dai, Yongqian Shu, Lin Xu, Zhibin Hu, Hongbing Shen

**Affiliations:** 1 Department of Epidemiology and Biostatistics, School of Public Health, Nanjing Medical University, Nanjing, China; 2 Jiangsu Key Lab of Cancer Biomarkers, Prevention and Treatment, Collaborative Innovation Center For Cancer Personalized Medicine, Nanjing Medical University, Nanjing, China; 3 Department of Thoracic Surgery, Affiliated Cancer Hospital of Nanjing Medical University, Jiangsu Key Laboratory of Molecular and Translational Cancer Research, Jiangsu Cancer Hospital, Nanjing, China; 4 Departments of Oncology, First Affiliated Hospital of Nanjing Medical University, Nanjing, China

## Abstract

Platinum-based chemotherapy has been shown to improve the survival of advanced non-small cell lung cancer (NSCLC) patients; the platinum-induced toxicity severely impedes the success of chemotherapy. Genetic variations, such as single nucleotide polymorphisms (SNPs), may contribute to patients’ responses to the platinum-based chemotherapy. To identify SNPs that modify the risk of hepatotoxicity in NSCLC patients receiving platinum-based chemotherapy, we performed a genome-wide association scan in 334 subjects followed by a replication study among 375 subjects. Consistent associations with platinum-induced hepatotoxicity risk was identified for SNP rs2838566 located at 21q22.3, as the minor A allele could significantly increase the risk of liver injury (OR = 3.78, 95%CI = 1.99–7.19, *P* = 4.90 × 10^−5^ for GWAS scan, OR = 1.89, 95%CI = 1.03–3.46, *P* = 0.039 for replication, and OR = 2.56, 95%CI = 1.65–3.95, *P* = 2.55 × 10^−5^ for pooled population). These results suggested that genetic variants at 21q22.3 may contribute to the susceptibility of platinum-induced hepatotoxicity in NSCLC patients.

Lung cancer is the leading cause of cancer-related mortality worldwide with about 1.38 million deaths estimated in 2008^1^. Approximately 80% of lung cancer cases are non-small cell lung cancer (NSCLC), which presents malignant behavior and poor prognosis with an estimated five-year survival rate at about 15%^2^,^3^. Platinum-based chemotherapeutic agents, such as cisplatin (DDP) and carboplatin (CBP), have been considered as the most effective treatment for advanced NSCLC^4^,^5^. However, the clinical use of platinum-based chemotherapy is often hampered by its severe side effects, such as nephrotoxicity, neurotoxicity and gastrointestinal toxicity^6^,^7^,^8^. Although platinum is a rare cause of hepatic toxicity such as steatosis and cholestasis at a standard dose^9^, the minor aspartate aminotransferase (AST) elevations are common^10^. Additionally, recent studies have suggested that hepatotoxicity is also a major dose limiting-factor when high dose platinum chemotherapy has been continued^11^,^12^. Oxidative stress plays a pivotal role as one of the most important mechanisms underlying platinum-induced hepatotoxicity^13^; however, the molecular mechanisms have not been fully characterized.

Several factors have been implicated in determining patients’ responses to chemotherapy toxicity, such as age, gender, drug administration schedule, and performance status^14^,^15^,^16^,^17^,^18^. In addition, patients’ genetic variations, such as single nucleotide polymorphisms (SNPs), may also play important roles in modulating response to therapy. Some studies have been conducted to explore the associations between the SNPs of several candidate genes and total platinum-induced toxicity or special toxicity like nephrotoxicity, ototoxicity and myelosuppression in NSCLC patients. For example, the associations between hsa-miR-196a2 rs11614913 and overall toxicity risk^19^, and CASP3 rs6948 and hematologic toxicity risk^20^ were observed. However, little is known about the effects of genetic susceptibility on the risk of hepatotoxicity induced by platinum-based chemotherapy in NSCLC patients.

Recently, genome-wide association studies (GWAS) have provided a robust tool to identify novel biomarkers for the development of complex traits by using high-throughput genotyping technology and selecting tagging SNPs across the whole genome^21^,^22^. In the present study, to comprehensively investigate the associations between SNPs and the susceptibility of platinum-induced hepatotoxicity of NSCLC patients in Chinese population, we carried out a GWAS scan by genotyping 906,703 SNPs in 334 NSCLC patients, followed by a replication in additional 375 NSCLC patients from Southeastern China.

## Results

The demographic and clinical characteristics of NSCLC patients in GWAS scan and replication study are shown in Table 1. Both stages had similar mean ages of patients (59.4 in GWAS and 60.1 in replication) and included more males (69.6% in GWAS and 67.5% in replication). The distribution of each characteristics was also similar between these two studies, with more smokers (55.0% in GWAS and 49.9% in replication), adenocarcinoma (70.2% in GWAS and 62.7% in replication), advanced stage (III and IV) (76.9% in GWAS and 65.9% in replication), surgical operation (50.5% in GWAS and 57.9% in replication), DDP- based chemotherapy (59.9% in GWAS and 56.3% in replication), and hepatotoxicity of grade 1 (53.2% in GWAS and 48.5% in replication).

**Figure 1:**
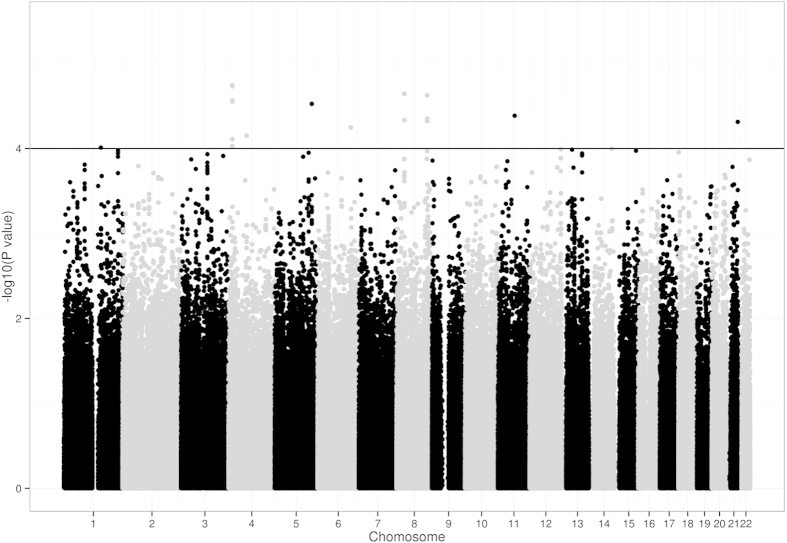
Genome-wide association results for platinum-induced hepatotoxicity in Han Chinese NSCLC patients. Scatter plot of P values in –log10 scale from GWAS results of the additive model on 588,732 SNPs.

*P* values for the discovery cohort were presented in the scatter plot with multiple suggestive associations (*P* < 1 × 10^−4^ in additive model; See Fig. 1). Eleven SNPs with *P* < 1 × 10^−4^ were selected for the validation while 9 other SNPs were excluded because of high LD with the selected ones (Table 2). In the replication stage, only the minor A allele of rs2838566 at 21q22.3 was found to be significantly associated with platinum-induced hepatotoxicity in the same direction with the GWAS scan (OR = 3.78, 95%CI = 1.99–7.19, *P* = 4.90 × 10^−5^ in GWAS scan, and OR = 1.89, 95%CI = 1.03–3.46, *P* = 0.039 in replication), compared with G allele (Table 3). When we pooled the subjects of GWAS and replication cohorts, rs2838566 was still associated with hepatotoxicity, with the *P* values being 2.55 × 10^−5^ (pooled OR = 2.56, 95%CI = 1.65–3.95). We then performed stratification analyses of rs2838566 in the pooled population to evaluate the effects of variant genotypes on the risk of platinum-induced hepatotoxicity by age, gender, smoking status, histology, stage, surgical operation, and platinum compounds (Table 4). The results showed that the association between rs2838566 and platinum-induced hepatotoxicity was significant in every stratum except among female-only populations. However, we didn’t observe any significant heterogeneity between each two stratums (*P* > 0.05) in (Table 4). The results for other 10 selected SNPs in replication phase and pooled population were shown in Supplementary Table S1 and Supplementary Table S2.

## Discussion

Platinum is widely used for the treatment of advanced NSCLC, which may induce cytotoxicity through its interaction with DNA to form DNA-protein and DNA-DNA interstrand crosslinks^23^. However, the effect of platinum is restricted because of its adverse effects, such as hepatotoxicity. Liver toxicity of platinum is mainly characterized by elevation of serum transaminases, bilirubin, and alkaline phosphatase. In this study, we represented the first GWAS on platinum-induced hepatotoxicity in NSCLC patients in Han Chinese population. With a two-stage design approach, we identified a locus at 21q22.3 (rs2838566) was significantly associated with liver toxicity in NSCLC patients receiving platinum-based chemotherapy.

The SNP rs2838566 is located in intergenic regions with some genes nearby, including *TRPM2* (transient receptor potential cation channel, subfamily M, member 2, 25 kb downstream), *C21orf2* (chromosome 21 open reading frame 2, 127 kb upstream), and *LRRC3* (leucine rich repeat containing 3, 11 kb downstream, (Supplementary Fig. S1 online). The TRPM2 channel protein encoded by *TRPM2* gene has two distinct domains with one function as an ion channel and the other as an ADP-ribose (ADPR)-specific pyrophosphatase^24^. The TRPM2 channel is also a redox-sensitive Ca^2+^-permeable cation channel, which is activated by several second messengers^25^,^26^,^27^, and is capable of mediating susceptibility to cell death^27^,^28^,^29^,^30^,^31^,^32^. Some studies have revealed that intracellular antioxidant or oxidant, such as glutathione (GSH), hydrogen peroxide (H_2_O_2_), and some toxins, could modulate Ca^2+^ influx and oxidative toxicity through TRPM2 channel^33^,^34^,^35^,^36^. Interestingly, oxidative stress plays an important role in the mechanisms underlying platinum-induced hepatotoxicity^13^, and we speculate that *TRPM2* may affect the susceptibility of liver injury through the oxidative stress response. Besides, some studies have investigated the role of TRPM2 in the development of human cancers. It was reported that selectively knocking down TRPM2 inhibited the growth of prostate cancer cells but not of non-cancerous cells^37^. *C21orf2* is a protein coding gene, and four alternatively spliced transcript variants encoding four different isoforms have been found for this nuclear gene. All isoforms contain leucine-rich repeats, and three of these isoforms are mitochondrial proteins. Shim KS *et al.* found that *C21orf2* was down-regulated in Down syndrome (DS) brain, which may represent mitochondrial dysfunction in DS patients^38^, while Cheon MS *et al.* pointed out that the expression level of *C21orf2* was increased in fetal cerebral cortex from DS patients at 18–19 weeks of gestational age^39^. *C21orf2* is also a compelling candidate gene in the pathogenesis of cone-rod dystrophy^40^. The protein C21orf2 was reported to show cancer-associated reactivity and reacted preferentially with serum from cancer patients, including colon, stomach, breast, and prostate cancers, compared with normal human serum, with regard to serological responses^41^. There are few researches exploring the function of LRRC3 gene or relevant protein in human disease thus far. Using SNPinfo Web Server (http://snpinfo.niehs.nih.gov/), we found that rs2838563 and rs4818719 located in the 3’-UTR of *LRRC3* have high LDs with rs2838566 (r^2^ = 0.911) and both can regulate the protein translation by affecting microRNA binding sites activity and transcription factors binding. Furthermore, with the Cancer Genome Atlas (TCGA) database (http://cancergenome.nih.gov/) , we found that *C21orf2* and *LRRC3* had lower expression levels in hepatic carcinoma (*P* = 0.049 and 2.29 × 10^−7^, respectively), and *TRPM2* had higher expression levels in hepatic carcinoma (*P* = 0.041), suggesting the importance of these genes in the development of hepatic disease. Besides, by the online tools of RegulomeDB (http://regulomedb.org) and TFSEARCH 1.3 (http://www.cbrc.jp/research/db/TFSEARCH.html), we found that rs2838566 may affect the binding of some transcription factors. Together, our results support that rs2383566 may contribute to the risk of platinum-induced hepatotoxicity in non-small cell lung cancer patients through regulating the transcription of several genes; however, the biological mechanism needs to be further studied.

Our study has a number of strengths. This is the first GWAS to investigate the susceptibility of platinum-induced hepatotoxicity in NSCLC patients. Additionally, we used the ordinal logistic model to maximize the use of information and used a two-stage study design to reduce the incidence of false positive. However, several potential limitations of the present study also warrant considerations. First, our sample size was relatively small, which may have limited statistical power. Second, exact biological mechanism of the promising variant could not be annotated and the real causal SNP was undetermined. Therefore, further studies with larger sample size and functional analysis are needed to validate and extend our findings.

## Methods

### Study populations

The NSCLC patients in discovery phase were a part from our previous GWAS on lung cancer susceptibility^42^. In this study, the subjects were restricted to those who received platinum-based chemotherapy, including cisplatin and carboplatin, for at least two cycles and had the full information of hepatotoxicity evaluation. Patients who accepted radiation or chemotherapy other than platinum-based drugs were excluded. Finally, 334 NSCLC patients from the Affiliated Cancer Hospital and the First Affiliated Hospital of Nanjing Medical University were selected for the analysis of hepatotoxicity in the discovery set. The replication study included 375 patients from Nanjing Thoracic Hospital, the Affiliated Cancer Hospital and the First Affiliated Hospital of Nanjing Medical University. Subjects in the discovery phase and the replication phase were all unrelated Han Chinese. All patients had histopathologically or cytologically confirmed NSCLC, which was reviewed by at least two local pathologists. Clinical data were systematically recorded at entry, including age at diagnosis, sex, smoking history, and family history of cancer, clinical stage, and tumor histology. Before starting the chemotherapy, all patients underwent a complete medical history interview, physical examination, and laboratory testing, including blood routine and biochemical examination. The liver function of all subjects was normal before chemotherapy according to liver function tests, as the value of bilirubin <17.1 μmol/L, alanine transaminase (ALT) and AST <40 U/L, and alkaline phosphatase <110 U/L. Those who had smoked less than 1 cigarette per day and less than 1 year in their lifetime were considered nonsmokers; all others were considered smokers. The demographic information was collected by face to face questionnaire investigations, and the clinical information was gathered from patients’ medical records.

### Ethics Statement

The research protocol was approved by the Institutional Review Board of Nanjing Medical University, and the study was carried out in accordance with the nationally approved guidelines. The study conformed to the ethical standards of the 1964 Declaration of Helsinki. And informed consent was obtained from each subject at the time of recruitment.

### Chemotherapeutic treatment and toxicity identification

All patients were treated with first-line platinum-based chemotherapy. Chemotherapeutic regimens included cisplatin/carboplatin/oxaliplatin/ nedaplatin plus gemcitabine (GP), cisplatin/carboplatin plus paclitaxel (TP), cisplatin/carboplatin plus docetaxel (DP), and cisplatin plus vinorelbine (NP). Three weeks (21 days) were considered as one cycle for above regimens. The chemotherapeutic protocol was as follows: cisplatin (75 mg/m^2^ on day 1) plus gemcitabine (1250 mg/m^2^ on day 1 and day 8); cisplatin (75 mg/m^2^ on day 1) plus paclitaxel (135 mg/m^2^ on day 1); cisplatin (75 mg/m^2^ on day 1) plus docetaxel (75 mg/m^2^ on day 1); carboplatin [area under curve (AUC) 5 on day 1] plus gemcitabine (1250 mg/m^2^ on day 1 and day 8); carboplatin (AUC 5 on day 1) plus paclitaxel (135 mg/m^2^ on day 1); carboplatin (AUC 5 on day 1) plus docetaxel (75 mg/m^2^ on day 1). Other regimens included cisplatin and vinorelbine, oxaliplatin and gemcitabine, nedaplatin and gemcitabine.

All patients received regular examinations during treatment, including routine blood test, liver and kidney function test, ECG and chest X-ray, to confirm a good chemo-toxicity tolerance. After rest period, these above treatments will be repeated. For all recruited patients, the treatment lasted from two cycles to six cycles. Patient charts were reviewed to check the information on experienced toxicities during the chemotherapy process. Complete medical records, including progress notes of the treating oncologist and treating nurses, chemotherapy infusion orders, and infusion flow sheets, were reviewed to collect these data. Specially, all the investigators were blinded to the polymorphism status of the patients.

Liver toxicity was assessed after 2 or 3 cycles of platinum treatment, according to the National Cancer Institute Common Terminology Criteria Adverse Events Version 3.0 (CTCAE v3.0, http://ctep.cancer.gov), and was classified into Grade 1 to 4 according to the peak value of bilirubin, ALT, AST, and alkaline phosphatase. The chemotherapy would be discontinued, postponed, or reduced in case of disease progression or unacceptable toxicity.

### Genotyping and Quality Control (QC)

Genotyping at the GWAS scan was performed using Affymetrix Genome-Wide Human SNP Array 6.0 chips. Before the genetic association analysis, we conducted systematic QC on the raw genotyping data to filter both unqualified samples and SNPs, as described previously^42^. SNPs were excluded if: (i) SNPs were not mapped on autosomal chromosomes; (ii) SNPs had a call rate <95%; or (iii) SNPs had minor allele frequency (MAF) <0.05. As for samples, two subjects were excluded as they showed gender discrepancies, one subject who were unexpected duplicates or probable relatives based on pairwise identity-by-state according to “PI_HAT” value in PLINK (all PI_HAT >0.25), and two cases seemed to be outliers in the principal component analysis using the software package EIGENSTRAT 3.0. Finally, 329 cases were used with 588,732 SNPs. The genotyping analysis for the replication subjects was done using the iPLEX Sequenom MassARRAY platform (Sequenom, Inc, CA, USA). The information on primers and probes are available upon request, and 5% of the samples were randomly selected for repeat genotyping and the concordance was 100%.

### Analysis Approach for Genetic Association

Genome-wide association analysis was performed in the additive model using ordinal logistic analysis with adjustment for age, gender, smoking status, histology, stage, and principal-component (the discovery phase only). Promising SNPs were defined if they had a *P* value less than 1 × 10^−4^. Finally, 11 SNPs were eligible and selected for further replication while 9 other promising SNPs were excluded because of high linkage disequilibrium (LD) with selected SNPs (r^2^ > 0.8).

### Statistical Analysis

We used PLINK 1.07 for general genetic statistical analysis^43^. The “rms” and “Rserve” package in R (PLINK plug-in) were used to perform the analyses of hepatotoxicity grade^43^,^44^. The ordinal logistic model was fit to the ordinal phenotype of hepatotoxicity grade levels^45^. Odds ratios (OR) and their 95% confidence intervals (CI) were calculated by multivariate logistic regression analyses and adjusted for age, gender, smoking status, histology, stage, and principal-component (the discovery phase only). The MACH 1.0 software (http://www.sph.umich.edu/csg/abecasis/MACH/index.html) was used to impute untyped SNPs by using 1000 Genomes database (http://www.1000genomes.org/46. Regional plot was generated using an online tool, LocusZoom 1.1 (http://csg.sph.umich.edu/locuszoom/). Analyses was also performed using SAS version 9.1.3 (SAS Institute, Cary, NC) or Stata version 9.2 (StataCorp LP, TX).

## Additional Information

**How to cite this article**: Cao, S. *et al.* Genome-wide Association Study on Platinum-induced Hepatotoxicity in Non-Small Cell Lung Cancer Patients. *Sci. Rep.* **5**, 11556; doi: 10.1038/srep11556 (2015).

## Supplementary Material

Supplementary Information

## Figures and Tables

**Table 1:** Demographic and clinical characteristics of study subjects.

Characteristics	GWAS(N = 329)	Replication(N = 375)
Age	59.4 ± 10.5	60.1 ± 9.3
Gender		
Male	229(69.6%)	253(67.5%)
Female	100(30.4%)	122(32.5%)
Somking status		
Never	148(45.0%)	166(44.3%)
Ever	181(55.0%)	187(49.9%)
Unknown	–	22(5.9%)
Histologic type		
Squamous cell carcinoma	98(29.8%)	98(26.1%)
Adenocarcinoma	231(70.2%)	235(62.7%)
Others^a^	–	42(11.2%)
Stage		
I~II	76(23.1%)	128(34.1%)
III~IV	253(76.9%)	247(65.9%)
Surgical operation		
No	163(49.5%)	158(42.1%)
Yes	166(50.5%)	217(57.9%)
Platinum compounds		
Cisplatin -based	197(59.9%)	211(56.3%)
Carboplatin -based	92(28.0%)	144(38.4%)
Other platinum^b^	40(12.2%)	20(5.3%)
Hepatotoxicity grade		
0 (No hepatotoxicity)	83(25.2%)	131(34.9%)
1	175(53.2%)	182(48.5%)
2	59(17.9%)	57(15.2%)
3~4	12(3.6%)	5(1.3%)

^a^Other carcinomas include large cell, mixed cell, or undifferentiated carcinomas.

^b^Other platinum compounds include oxaliplatin(L-OHP) and nedaplatin(NDP).

**Table 2:** The associations between 11 selected SNPs from GWAS scan and hepatotoxicity risk.

Location	SNP	Allele	Genotype	MAF^a^	HWE^b^	OR(95% CI)^c^	*P*^c^
8p12	rs16878272	C > G	144/142/43	0.344	0.281	0.51(0.37,0.70)	2.27 × 10^−5^
8q24.13	rs13267737	G > A	195/115/19	0.233	0.759	2.16(1.51,3.08)	2.37 × 10^−5^
5q33.2	rs17053350	C > T	108/168/43	0.397	0.083	2.03(1.46,2.83)	2.98 × 10^−5^
11q13.4	rs947853	C > T	146/138/45	0.349	0.228	0.52(0.38,0.71)	4.11 × 10^−5^
8q24.13	rs7008590	G > A	202/105/22	0.227	0.160	2.08(1.46,2.95)	4.47 × 10^−5^
21q22.3	rs2838566	G > A	290/37/2	0.062	0.352	3.78(1.99,7.19)	4.90 × 10^−5^
6q23.3	rs9402873	C > T	285/40/4	0.073	0.077	3.37(1.86,6.08)	5.65 × 10^−5^
4q13.3	rs4446279	G > C	177/127/25	0.269	0.889	2.00(1.42,2.82)	7.07 × 10^−5^
4p15.32	rs4140932	T > A	103/166/60	0.434	0.738	1.87(1.37,2.55)	7.79 × 10^−5^
4p15.32	rs13131227	A > G	135/153/41	0.356	0.905	1.87(1.37,2.57)	9.41 × 10^−5^
1q23.1	rs6681909	C > G	199/106/17	0.218	0.630	2.06(1.43,2.97)	9.75 × 10^−5^

^a^Minor allele frequency (MAF).

^b^Hardy-Weinberg equilibrium (HWE).

^c^Odds ratio and *P* value of ordinal logistic analysis in additive model, adjusted for age, gender, somking status, histologic type ,stage and principal-component.

**Table 3:** Association between rs2838566 genotypes and risk of platinum-induced hepatotoxicity in different stages.

	Genotype (Grade 0/1/2/3~4)	OR(95%CI)^a^	*P*^a^	OR(95%CI)^b^	*P*^b^
**GWAS scan**					
GG	78/160/45/7	1.00		1.00	
AG	4/15/14/4	**4.10(2.09, 8.01)**	**3.63 × 10**^**−5**^	**4.08(2.08,7.99)**	**4.31 × 10**^**−5**^
AA	1/0/0/1	3.65(0.03,443.45)	0.597	4.08(0.04,392.67)	0.546
dominant	–	**4.01(2.07,7.78)**	**3.96 × 10**^**−5**^	**4.01(2.06,7.79)**	**4.31 × 10**^**−5**^
additive	–	**3.80(2.00,7.21)**	**4.31 × 10**^**−5**^	**3.78(1.99,7.19)**	**4.90 × 10**^**−5**^
**Replication phase**					
GG	110/151/46/5	1.00		1.00	
AG	9/17/4/0	1.08(0.54,2.16)	0.829	1.22(0.60,2.48)	0.585
AA	0/0/3/0	**19.30(2.51,147.82)**	**0.004**	**22.86(2.74,190.62)**	**0.004**
dominant	–	1.40(0.71,2.74)	0.330	1.59(0.80,3.17)	0.183
additive	–	1.68(0.92,3.05)	0.091	**1.89(1.03,3.46)**	**0.039**
**Pooled analysis**					
GG	188/311/91/12	1.00		1.00	
AG	13/32/18/4	**2.20(1.35,3.58)**	**0.002**	**2.34(1.43,3.82)**	**0.001**
AA	1/0/3/1	**13.10(2.25,76.30)**	**0.004**	**13.43(2.26,79.66)**	**0.004**
dominant	–	**2.43(1.51,3.91)**	**2.62 × 10**^**−4**^	**2.60(1.59,4.14)**	**1.04 × 10**^**−4**^
additive	–	**2.44(1.58,3.77)**	**5.82 × 10**^**−5**^	**2.56(1.65,3.95)**	**2.55 × 10**^**−5**^

^a^Crude odds ratio and *P* value of ordinal logistic analysis.

^b^Odds ratio and *P* value of ordinal logistic analysis, adjusted for age, gender, somking status, histologic type ,stage and principal-component (GWAS scan only).

**Table 4:** Stratification analysis of rs2838566 genotypes associated with platinum-induced hepatotoxicity in pooled NSCLC patients.

Charactersitcs	Genotype(GG/AG/AA)				OR(95%CI)^a^	*P* for heterogeneity
	Grade 0	Grade 1	Grade 2	Grade 3–4		
Age						
≤60	89/5/0	154/17/0	57/11/1	9/0/0	2.44(1.30,4.58)	0.868
>60	99/8/1	157/15/0	34/7/2	3/4/1	2.63(1.42,4.88)	
Gender						
Male	132/8/1	214/21/0	60/13/3	7/3/0	2.83(1.68,4.76)	0.577
Female	56/5/0	97/11/0	31/5/0	5/1/1	2.12(0.94,4.76)	
Somking status						
Never	78/6/0	137/12/0	46/10/1	8/1/1	2.60(1.35,4.99)	0.951
Ever	107/6/1	162/20/0	42/8/2	3/3/0	2.53(1.41,4.56)	
Histologic type						
Squamous cell carcinoma	64/3/1	81/11/0	19/6/0	2/1/0	2.46(1.04,5.79)	0.718
Adenocarcinoma	112/8/0	213/20/0	66/12/3	9/3/1	2.96(1.76,4.99)	
Stage						
I~II	65/3/0	83/9/0	24/3/2	3/1/0	2.80(1.26,6.22)	0.804
III~IV	123/10/1	228/23/0	67/15/1	9/3/1	2.48(1.46,4.22)	
Surgical operation						
No	75/8/0	143/20/0	51/6/2	7/2/1	2.67(1.48,4.84)	0.633
Yes	113/5/1	168/12/0	40/12/1	5/2/0	2.15(1.11,4.18)	
Platinum compounds						
DDP	112/9/1	176/21/0	50/10/0	6/2/1	2.18(1.19,3.99)	0.225
CBP	62/4/0	103/11/0	33/6/3	5/2/0	2.79(1.45,5.37)	
Other platinum^b^	14/0/0	32/0/0	8/2/0	1/0/0	31.51(1.53,649.47)	

^a^Odds ratio of ordinal logistic analysis in additive model, adjusted for age, gender, somking status, histologic type and stage.

^b^Other platinum compounds include oxaliplatin(L-OHP) and nedaplatin(NDP).
