# Supplementary material for: Genome-wide Association Study on Platinum-induced Hepatotoxicity in Non-Small Cell Lung Cancer Patients
Source: Sci Rep. 2015 Jun 23;5:11556. doi: 10.1038/srep11556 (PMC4477405; doi:10.1038/srep11556)
Supplement: Supplementary Information [file srep11556-s1.pdf]

Genome-wide Association Study on Platinum-induced Hepatotoxicity in Non-Small Cell Lung Cancer Patients

Songyu Cao[1#], Cheng Wang[1,2#], Hongxia Ma[1,2], Rong Yin[3], Meng Zhu[1], Wei Shen[1], Juncheng Dai[1,2], Yongqian Shu[4], Lin Xu[3], Zhibin Hu[1,2], Hongbing Shen[1,2*]

[1]Department of Epidemiology and Biostatistics, School of Public Health, Nanjing Medical University, Nanjing, China;

[2]Jiangsu Key Lab of Cancer Biomarkers, Prevention and Treatment, Collaborative Innovation Center For Cancer Personalized Medicine, Nanjing Medical University, Nanjing, China;

[3]Department of Thoracic Surgery, Affiliated Cancer Hospital of Nanjing Medical University, Jiangsu Key Laboratory of Molecular and Translational Cancer Research, Jiangsu Cancer Hospital, Nanjing, China;

[4]Departments of Oncology, First Affiliated Hospital of Nanjing Medical University, Nanjing, China.

[#] These authors contributed equally to this work.

***** To whom correspondence should be addressed at: Department of Epidemiology and Biostatistics, School of Public Health, Nanjing Medical University, No. 101 Longmian Road, Nanjing 211166, China. Email: hbshen@njmu.edu.cn.

Supplementary Table 1 Association of other 10 selected SNPs with platinum-induced hepatotoxicity in replication phase

SNP	Genotype[a]	MAF[b]	HWE[c]	OR(95% CI)[d]	*P*[d]
rs9402873	287/58/2	0.088	0.628	0.66(0.40,1.11)	0.115
rs17053350	142/157/48	0.366	0.868	0.85(0.63,1.14)	0.280
rs947853	138/162/50	0.377	0.924	0.87(0.64,1.18)	0.380
rs13131227	148/165/31	0.327	0.129	1.16(0.83,1.61)	0.396
rs4140932	105/147/67	0.565	0.206	1.13(0.84,1.53)	0.418
rs7008590	205/123/18	0.228	0.944	0.89(0.63,1.26)	0.516
rs16878272	136/155/53	0.380	0.401	1.10(0.82,1.49)	0.526
rs6681909	225/115/12	0.196	0.562	0.91(0.63,1.32)	0.608
rs13267737	203/123/21	0.239	0.779	0.95(0.68,1.32)	0.751
rs4446279	173/136/39	0.691	0.114	1.02(0.75,1.39)	0.876

[a]Frequency of wild homozygous, heterozygote and variant homozygous of these SNPs.

[b]Minor allele frequency (MAF)

[c]Hardy-Weinberg equilibrium (HWE)

[d]Odds ratio and *P* value of ordinal logistic analysis in additive model in replication phase, adjusted for age, gender, smoking status, histologic type and stage.

Supplementary Table 2 Association of other 10 selected SNPs with platinum-induced hepatotoxicity in pooled population

SNP	Genotype[a]	MAF[b]	HWE[c]	OR[d]	*P*[d]	OR[e]	*P*[e]	OR(95% CI)[f]	*P*[f]
rs947853	284/300/95	0.140	0.272	0.52	4.11×10^{-5}	0.87	0.380	0.68(0.55,0.84)	3.57×10^{-4}
rs13131227	283/318/72	0.107	0.213	1.87	9.41×10^{-5}	1.16	0.396	1.49(1.19,1.86)	0.001
rs4140932	208/313/127	0.196	0.636	1.87	7.79×10^{-5}	1.13	0.418	1.41(1.14,1.75)	0.002
rs4446279	350/263/64	0.095	0.158	2.00	7.07×10^{-5}	1.02	0.876	1.36(1.09,1.71)	0.007
rs13267737	398/238/40	0.059	0.310	2.16	2.37×10^{-5}	0.95	0.751	1.38(1.09,1.76)	0.008
rs16878272	280/297/96	0.143	0.232	0.51	2.27×10^{-5}	1.10	0.526	0.76(0.61,0.93)	0.009
rs6681909	424/221/29	0.043	0.976	2.06	9.75×10^{-5}	0.91	0.608	1.40(1.08,1.81)	0.010
rs7008590	407/228/40	0.059	0.288	2.08	4.47×10^{-5}	0.89	0.516	1.35(1.06,1.72)	0.016
rs17053350	250/325/91	0.137	0.367	2.03	2.98×10^{-5}	0.85	0.280	1.28(1.03,1.59)	0.028
rs9402873	572/98/6	0.009	0.433	3.37	5.65×10^{-5}	0.66	0.115	1.28(0.86,1.89)	0.220

[a]Frequency of wild homozygous, heterozygote and variant homozygous of these SNPs.

[b]Minor allele frequency (MAF)

[c]Hardy-Weinberg equilibrium (HWE)

[d]Odds ratio and *P* value of ordinal logistic analysis in additive model in GWAS scan, adjusted for age, gender, smoking status, histologic type, stage and principal-component.

[e]Odds ratio and *P* value of ordinal logistic analysis in additive model in replication phase, adjusted for age, gender, smoking status, histologic type and stage.

[f]Odds ratio and *P* value of ordinal logistic analysis in additive model among pooled population, adjusted for age, gender, smoking status, histologic type and stage.

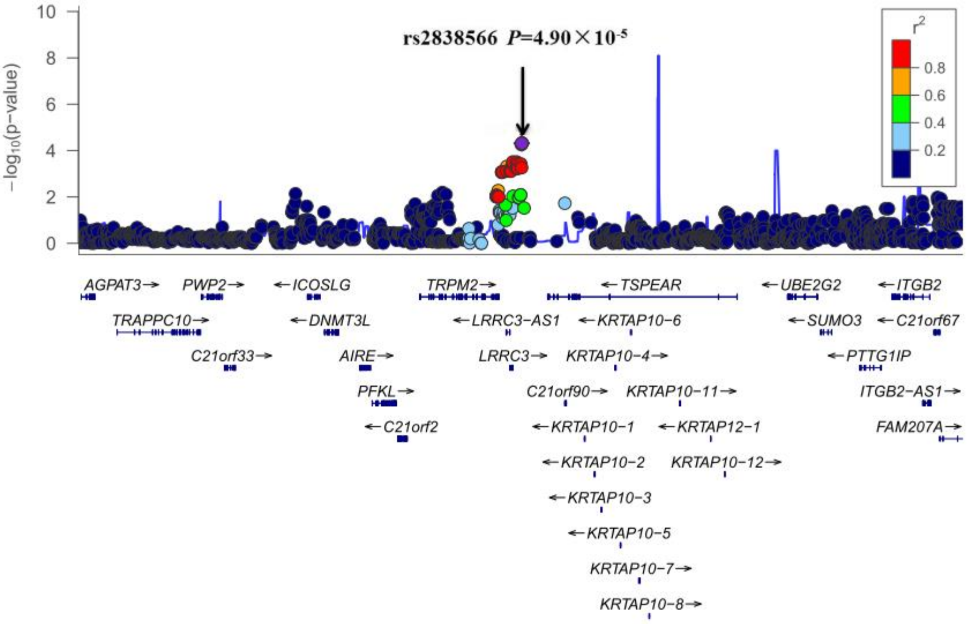

Supplementary Figure S1. Regional plot of rs2838566. Result (-log 10 P) are shown in the region flanking 500 kb on either side of rs2838566. Rs2838566 was shown in purple diamond and the R^2 values of the rest of the SNPs are indicated by different colors.
